# 学習環境を動的なシステムとして捉え直す

**DOI:** 10.12688/f1000research.153938.1

**Published:** 2024-10-10

**Authors:** 伊藤秀明 伊藤

**Affiliations:** 1 University of Tsukuba, Tsukuba, Ibaraki Prefecture, Japan

**Keywords:** xperiential-ecological learning environments, learning environment engineer, environmental enrichments, affordance, meaningful valuing

## Abstract

日本語教育において「学習環境」という用語はさまざまな文脈で用いられている。そこで本稿では、先行研究の「学習環境」という用語の使用実態を調査し、従来の学習環境は静的な学習者を中心にして、その特徴を捉えた「形態特徴的学習環境」であることを述べた。そして、学習環境は学習主体と環境とが相互作用をしながら変化していく能動的な循環関係であることから、動的な学習者/学習環境の姿を捉えようとする「経験・生態的学習環境」へと学習環境の捉え直しをしていくことを主張した。その上で、学習主体の経験・生態的学習環境を豊かにしていくためには、学習環境エンジニアの存在および意味のある価値づけとなる環境エンリッチメントの配置が重要であることを述べた。さらに個別の学習環境だけではなく、さまざまな人々の経験を組み合わせていくことで、持続的かつより豊かな学習環境を構築していくことができることを強調している。

## Abstract (en)

In Japanese language education, the term "learning environment" is used in various contexts. This paper investigates the term "learning environment" in previous studies and notes that traditional learning environments have been characterized as "morphological characteristics learning environments" focusing on static learners. However, since learning environments involve a dynamic, reciprocal relationship where learners and their surroundings interact and evolve, the paper advocates for a re-conceptualization of "experiential-ecological learning environments" that captures this dynamic nature. Furthermore, to enrich the experiential-ecological learning environment for learners, the presence of learning environment engineers and the meaningful valuing of environmental enrichments is essential. Lastly, the paper emphasizes that by combining various individual learning experiences, it is possible to create a sustainable and richer learning environment.

## 1. はじめに

本稿は，日本語教育の先行研究においてさまざまな文脈で用いられてきた「学習環境」という用語が，これまでどのように捉えられてきたのかを検討し，「学習環境」に対する捉えを静的なものから動的なものに捉え直すことで，学習環境を動的なシステムとして考えていく必要性を主張することを目的とする。

日本語教育において「学習環境」という用語は日本国内/国外，またはそこに付随する漢字圏/非漢字圏のように「学習者の居住地の特徴」を述べたり，教室環境/自然環境のように「学習者が日本語を学ぶ（習得する）場所」という文脈で用いられたり，対面/オンラインのように「学習者の学習形態」を指すなど，その用いられ方は多岐にわたる。同様に， 文化庁（2024 年度より文部科学省へ移管）が進めている「『生活者としての外国人』のための日本語教室空白地域解消推進事業」の目的では「本事業は，日本語教室が設置されていない国内の地域に居住している外国人等が，日常生活を営む上で必要となる日本語能力を習得できるよう，日本語教室の設置，ICT を活用した日本語学習コンテンツの開発等を行うことにより， 日本語学習環境の整備を図ることを目的としています。」（下線は引用者による）と「日本語教室の設置」や「ICT を活用した日本語学習コンテンツの開発」という物理的環境の整備が日本語学習環境の整備であるように述べられている一方， 国際交流基金は 2022 年度の年報の「海外における日本語教育」において「海外の人たちに日本語を知ってもらうことは，日本への親しみや理解を広げるきっかけとなります。世界中で多くの人に日本語を学んでもらえるよう，各国の 日本語学習環境の整備を進めています。」 (p.10) （下線は引用者による）と述べ，それらを進める事業として専門家派遣や教師研修などを挙げているように，行政機関においても「学習環境」という語の使用は見られるものの，「学習環境」が何を指しているのかは明確に示されてはいない。このようにさまざまな文脈で用いられる「学習環境」という語は先に述べたように，日本国内/国外，漢字圏/非漢字圏のように二項対立で分けられることも多く，一方の優位性や不十分さという学習環境の上下関係を作り出すレトリックとして発信者に使用されることもある。これらのことを踏まえ，「学習環境」とはどのようなものであるのかを明確にしていく必要がある。

日本語教育における「学習環境」という用語の初出は，国立情報学研究所が提供するデータベース「CiNii Reserch」で「学習環境 and 日本語教育」を検索すると，1989 年から始まる科学研究費補助金の研究概要において確認できる。その後，1990 年代半ばから論文等でも使用されることが多くなっている。近年では社会的な変化により対面環境/オンライン環境という言葉がよく聞かれるようになっており，「学習環境」が指し示す意味も従来の意味とは変化している可能性もある。そのため，従来の「学習環境」が示していたものがどのようなものであるかを確認し，現在の状況に合わせた日本語教育における「学習環境」の捉え直しの必要性を検討していく。

## 2. 本研究で行なった調査概要

現在の状況に合わせた日本語教育における「学習環境」の捉え直しの必要性を検討するために，日本語教育に関する過去の文献において「学習環境」という用語がどのように使用されてきたのかを調査する。そこで本研究では以下のような調査を実施した。国立情報学研究所が提供しているデータベース「CiNii Research」において「学習環境 and 日本語教育」で検索し，データ種別が「論文」となっているものを抽出した。2024 年 4 月 2 日現在 ^1^，上記方法で抽出できた論文は 75 件（重複1件除外）であり，そのうち論文原稿を確認できたものは 62 件であったため，この 62 件すべてを本調査の対象とした。そして調査対象とした論文 62 件すべてに対し，各論文において「学習環境」という用語がどのような観点で使用されているのかを1件ずつ読み，確認した。調査対象論文の中にはキーワードのみで具体的な記述がないものもあったため，具体的な記述があったものを中心に「学習を行う物理的な場」「学習者の周りに配置するリソース」「授業内で行う学習活動の多様性，心理的側面への配慮」「自らが作り出すもの」の 4 つの観点に分類した。なお，62件の論文は文献リスト ( Ito, 2024) に挙げている。

## 3. 学習環境はどのように捉えられてきたのか

本節では「学習環境」という用語の使われ方によって分類した「学習を行う物理的な場」「学習者の周りに配置するリソース」「授業内で行う学習活動の多様性，心理的側面への配慮」「自らが作り出すもの」の観点ごとに，対象論文内でどのように述べられていたかについて触れる。

### 3-1 学習を行う物理的な場としての学習環境

漢字学習に対する学習者の信念は使用教科書や学校の所在地によって異なるのかを調査した 大北 (1998) は「文字導入の時期の信念に対しては教科書よりも場所の影響が強いと考えられる」(p.43)，「教科書よりも学習環境が会話学習優先信念に与える影響が大きいことを示唆している」(p.43)と「学習環境」を地理的環境，日本人との接触頻度などの日本語のインプットの多少として捉えている。また， 菅 (2017) では，日本語スピーチコンテストを通した日本語学習支援において，大教室にステージが整備されたことによりコンテスト後も声を出して練習できる新しい学習環境が形成されたと述べており，ステージがある教室を学習環境として捉えていることがわかる。 福島・マリーナ (2006) は教室外で日本語との接触機会がない海外環境における日本語学習環境を「孤立環境における日本語教育」と呼び，その地理的特徴を述べ， ショリナ (2019) は 福島・マリーナ (2006) を参考に，ある地域の学習環境を捉える際に，学習環境を社会的要因（日本との地理的・文化的な距離）と情意的要因（その社会においての日本語の実用性）に分けて捉える必要性を述べている。ただし，情意的要因も社会的要因に影響されることから ショリナ (2019) も学習環境は物理的な場として捉えていると考えられる。また， 立間 (2010) も 福島・マリーナ (2006) が述べる孤立環境で学習環境をどうデザインするかを問題意識として，日本とアゼルバイジャンの関係，日本企業数，在留邦人数，日本語学習者数など日本や日本人との社会的接触環境を調査していることから物理的環境として学習環境と捉えていることがわかる。その他，学習環境について触れている論文で最も多い言語習得に関する論文（ 伊藤，2005; 高・崔，2023; 辛，2005, 2006; 孫，2008a b; 東，2007; 村田，2020; 李，2003）では，学習環境として JSL/JFL 環境で調査対象者を分類しており，これらの論文では上で述べてきたことと同様に，学習環境を「物理的な場」として捉えている。

### 3-2 学習者の周りに配置するリソースとしての学習環境

羽田野・田口 (1995) では，高等学校における日本語教育の現場について「学習環境」という項を立てて取り上げている。そこでは「まず，学習理解のための日本語能力を高める適切な教材がない。また，日本語教育の専門教育を受けた教師はほとんど存在しない。そのため，日本語教育を必要とする生徒を入学させた場合は，教師が何の知識もないまま，手探りで対応することになる。また，定時制では時間割の関係から，日本語教育に割り当てられる時間が限られる。」(p.42)と教材や教師というリソースの有無や学習時間，学習上の制約など学習の前提となる環境を「学習環境」と捉えている。本調査の対象論文を年代順に並べた時に論文のキーワードとして初めて「学習環境」という用語を記載した 金田ほか (1997) では，「近年，日本語を学習する外国人が増加しているが，学習者が手軽に学べる教材や，二ヶ国語の話せる教師が不足しており，日本語学習環境はあまり整っていない」(p.46)と，教材や教師の存在の不足を「学習環境」が整っていない状況と捉えている。 来嶋ほか (1995) は多読を進めるために辞書機能のある読書支援プログラムの開発研究において課題として「学習環境」を挙げ，「コンピュータの台数を増やし，機種を向上させて利用しやすい学習環境をつくること」(p.11)と施設としての「物理的リソース」を学習環境と捉えている。また， 戸田 (2009) も「教室内外において発音練習ができる学習環境を整備し，学習機会を提供することにより，自律学習を促していく」(p.56)ということを提案し，その提案の具体例として「シャドーイング練習用DVD教材」「オンデマンド日本語発音講座」「日本語発音練習用ソフトウェアの開発」といずれも「物理的リソース」を挙げている。このように物理的リソースを学習環境と捉えるのは，漢字教育への CAI の導入について報告し，その利点と課題の検討を行なった 鈴木 (1995, 1996) も同様で，コンピュータの台数の不足に加えて，教育用ソフト（教材）の充実という物理的リソースが学習環境の中心に捉えられている。この流れは ICT の活用が進むにつれ増えていき， 来嶋・鈴木 (2000) では独習を支援する学習環境として「留学生は，正規の日本語の授業を離れた後も独自に日本語力を向上させる努力を続けており，新聞やテレビ，日本人の友人など，容易に接することのできるメディアを利用している。留学生の生活は，専門分野の研究やアルバイトなどで，教師が想像するよりも多忙である。したがって，彼らの独習を支援するためには，独習用教材も上記のメディアと同様，個人で容易に利用できることが求められる。開発と実践にあたって，このような状況を前提とし，少なくとも，時間や場所が自由に選べるなどの十分な配慮をすることが必要である」(p.9)と述べ，コンピュータの利用台数が限定的であったことから好きな時間と場所で利用できないという課題に対する提案として，友人なども含めて時間や場所が自由に選べるなどの配慮の必要性を述べている。このことから学習環境を「学習者の周りに配置するリソース」として捉えていると考えられる。これは 田中ほか (2005)， 沖本 (2019)， 深田・高橋 (2021) でも同様である。さらに， ハリソン・實平 (2005, 2007) では「学習環境設計」という用語を用いて，CALL (Computer Assisted Language Learning) 環境を前提として机の配置やメディアなど教室設計についても詳しく述べていることから「学ぶ場所，学ぶリソース」を一括して学習環境として捉えている。

### 3-3 授業内で行う学習活動・心理的側面への配慮としての学習環境

日系ブラジル人とその配偶者を対象に日本語の読み書き能力に関する調査をおこなった 野元 (1999) では，「外国人住民にひらがな・カタカナの読み書きのできない人が多いという事態が生じるのは，ひらがな・カタカナ習得の機会が不足しているという学習環境に原因があるというよりは，ひらがな・カタカナは漢字かなまじりで使用されることが多く，漢字のわからない人にとって，その有用性が現状では極めて低いことと関連深い」(p.440)と学習環境を「日本語習得の機会」として捉えている。 金庭 (2004) は学習環境を論文のキーワードとして挙げつつも，本文では「学習環境」という語を用いてはいないが論文の冒頭に「近年，学習者の日本語能力を高めるために，日本語クラスと地域社会との間に関連性を持たせることが課題となっている。」(p.4)と述べ，インターネットを活用して地域社会の情報を授業に導入する方法を提案していることから，「学習活動」が学習環境であると捉えていると考えられる。そして， 大石ほか (2005) では終日一教員担当制による利点を主張し，その中で「一教員制は，コマ割りの授業による学習者の思考の寸断を防ぎ，学習環境を安定させることからも有用である。」(p.5)と「授業内の活動」を学習環境と捉えていることがわかる。また， 大関・遠藤 (2012) は「他者と関わりながら学習に参加すること，つまり，協働的な学習環境が主体的な学びを支え」(p.73)と，「他者と関わりながら学習に参加する」という「活動形態」を学習環境と捉えている。この点について 広瀬 (2015) も「協働的学習環境」という語を用いて同様のことを述べている。

予備教育生の多様化が起こる中で学習者のニーズ分析をおこなった 小川ほか (1998) も学習環境とは何かという具体的な記述はないが，1) 留学の目的，動機，予想（期待）に関するもの，2) 留学生の心理的側面に関するもの，3) 言語学習観と他の外国語学習経験の影響を分析・考察し，学習者の傾向や変化を明らかにすること，がよりよい学習環境作りの一助になると期待しており，その点で目的や動機，異文化の中での心理的負担，言語学習に関する考え方という「心理的側面への配慮」が学習環境を整えると考えていることが窺える。 野原 (1999) は学習者の自発的な発話を促すために教師はどのような支援をしていくかを考察し，「教師に求められている学習支援的な役割とは，偏に，『言語習得を促進するような学習環境を整えること』と言える」(p.102)と述べた上で，その学習環境を「視聴覚機材等の設備面のことではなく，学習者が授業に興味を持って積極的に参加できるような場としての環境」(p.102)と 3-2節で述べた物理的リソースとしての学習環境をはっきりと除外し，「学習者の心理的側面に配慮した場」として捉えている。また， 秋元ほか (2016) では視覚に障害を持つ日本語学習者の学習環境を調査するにあたって，教師視点として教育現場が抱える不安や困難さを聞き取る調査を行なっており，学習者本人だけではなく，「学習者の周りの人の心理的側面」も含めて学習環境と捉えている。

### 3-4 自らが作り出すものとしての学習環境

ニーズ分析の重要性を述べている 若林 (1999) は，「ニーズ分析をすることで学生の学習環境を見直すことができると思う。主観的ニーズは現在行われている授業の中から生まれた学習者の要求であるから，ニーズ分析によって独善的になりがちな授業をもう一度見直す機会が教師に提供されているのである。しかし教師自身に学生が何を求めているか，またそれに適した学習環境を作るという考えがないなら，学生のニーズは何も見えてこないだろう」(p.80)と，「学習環境を見直す」「教師自身に学生が何を求めているか，またそれに適した学習環境を作るという考え」「それぞれの学習環境に適した効果的学習環境を作っていかなければならない」と「学習環境」は自由に変えていけるものであると捉えている。また，「ニーズ分析を基に，学習者と継続的な話し合いを通じて，個々の学生にとって最適な学習環境を作っていく過程の手伝いができ，さらに将来的にはその学生が授業外でも，あるいは卒業してからでも，日本語の自律学習，生涯学習ができるようになると考えられる」と，自らが学習環境を作っていくという自律的な学習環境の構築につながる考え方も提示しており， 3-1 節， 3-2 節， 3-3 節に挙げた「学習環境」とは異なる捉え方を提示している。 高橋・向井 (2006) も教室活動を通して日本語を教える者は，毎日の生活の中で日本語学習が行われていることを忘れがちであると指摘し，現実の学習環境の実態を把握し，学習を環境との相互作用の中で捉えていく必要性を述べている点で，学習環境を「自らが作っていくもの」として捉えていることが窺える。これは 向井・高橋 (2007) も同様の捉え方をしている。さらに， トムソン木下 (2007) は学習環境を「学習者コミュニティー」として捉え，Engeström の活動理論に当てはめて分析している。その中で「学習者コミュニティーには，達成すべき目標があり，成員は様々な役割を担い，目標に向かって協働していく。そこにはおのずとコミュニティーの文脈が蓄積され，慣習や固有の“文化”が発生し，短いながらも“歴史”も共有されていく。この“文化”“歴史”という言葉も社会文化理論の専門用語的な用法である。一般に言う日本の文化，日本の歴史などの用法とは一線を引き，あくまでもコミュニティーの中で生まれたものを指し，微視的なものも含む。例えば，教室内で人々が一定の行動や慣習を共有したら，それは教室の"文化"であり，一分前の出来事でも，時間軸を追って蓄積されることで次の出来事に影響を及ぼすなら，これを"歴史"と捉える。」と述べており，若林 (1999) は将来的な視点のみだったものに加えて，過去の視点も取り入れている。また， 滑川 (2015) は 岡崎 (2009) の「ある人のことばの力が存分に発揮される社会的環境（生態）がその人の生活の質を規定すると捉える」(p.12)という言語生態学の視点から学習環境を捉えている。

## 4. 学習環境の捉え直し

ここまで見てきたように，これまでの「学習環境」は「学習を行う物理的な場」「学習者の周りに配置するリソース」「授業内で行う学習活動の多様性，心理的側面への配慮」「自らが作り出すもの」とさまざまな観点から述べられている。個々の観点は学習環境を構成する重要なものである。しかし，「学習環境」を述べる観点がそれぞれの研究の文脈に寄りすぎており，個々に異なるものを述べているようにも見える。これは従来の「学習環境」の捉えにおいて当初予想していた年代における偏りが見られない点からも，学習者を中心に据えながらも学習者を無機質な固定化された存在として捉え，「学習者の環境を研究者がどう捉えるか」という個々の研究者による「形態特徴的学習環境」の捉えが中心にあるからではないだろうか（ 図1）。

**図1.**
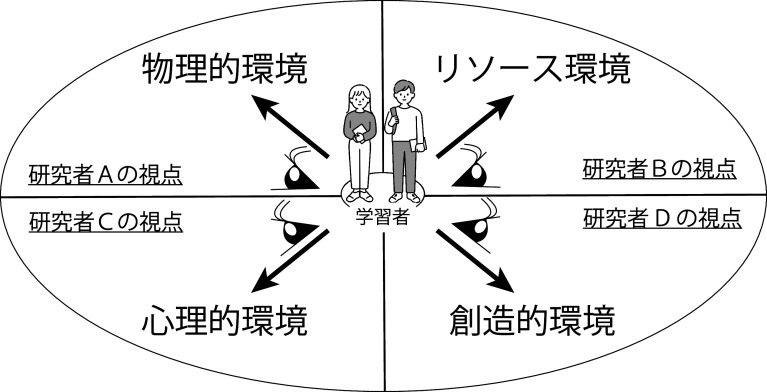
形態特徴的学習環境の捉え.

この形態特徴的学習環境の捉えの欠点としては①学習者を取り巻く環境が相互作用によって生み出される動的なものであるという視点が抜け落ちていること（動的システムとしての学習環境の視点の欠如），②学習者や学習者の周りにいる人々を環境に変化をもたらさない無機質な存在として捉えていること（学習環境エンジニアとしての存在/役割の軽視）の 2 点が考えられる。このような形態特徴的学習環境の捉えの欠点はどのようにすれば乗り越えられるのか。それには，日本語学習者の学習環境の特徴を捉えた形態特徴的学習環境としての捉えから，日本語学習者の経験によって変化する学習環境のあるがままを捉えた「経験・生態的学習環境」への捉え直しが重要であると考える。次節では，動的システムとして機能する「経験・生態的学習環境」とそれを創り出す「学習環境エンジニア」と「学習環境エンリッチメント」について詳しく述べる。

### 4-1 動的システムとしての経験・生態的学習環境

田中・斎藤 (1993) が「学習過程とは自分の身のまわりの環境になんらかのしかたで働きかけ，その環境を変えながら自らも変わっていく活動」と述べているように，学びにおいて学習主体と環境は相互作用をしながら変化していく。そして人間や社会は絶えず変化している点を踏まえると，それは単にお互いに影響を与え合っているというレベルに留まらず，さまざまな出来事を通した経験によって学習主体自身の状況認知が変化すると同時に，その状況認知の変化によって学習主体の学習環境が変化し，その学習環境の変化によって，さらに学習主体が変化していくという能動的な循環関係になっていると考えるべきである。そして，そのような関係において 図1 で示したような静的な形態特徴的学習環境の捉えでは限界があり，この動的な学習環境の姿を捉えようとするのが「経験・生態的学習環境」の捉えである。その意味で，経験・生態的学習環境は形態特徴的学習環境の捉えの欠点である「動的システムとしての学習環境の視点の欠如」を乗り越えるものである。

経験・生態的学習環境について考えるには，人類がどのように進化を遂げてきたのか，つまり，どのように複雑な社会を生き抜き，より良く生きようとしてきたのか，まで視点を広げる必要がある。 トマセロ(2022/2023) では，神経系を備えた人類の祖先から人類までの大まかな進化の流れについて左右相称動物→脊椎動物→哺乳類→大型類人猿→人類との仮説を立て，行為主体が意思決定を行い，能動的に行動する「行為主体性の変化」の流れについて論じている。そして，生物種が抱く「目標や基準値は，追求されるべき事実的な状況として表象され」(pp.62-63)，行動の意思決定は生物の目標に応じて決まり，その際の注意の対象となるのは自己の目標や行動に関連する状況であると述べている。 ユクスキュル・クリサート(1970/2005) は，このように生物種が自身を取り巻く環境から主観的に意味を構築して創り出して見ている世界を「環世界 (Umwelt)」 ^2^と呼び，それぞれの有機体が同一の物理的環境の中で異なる世界（環世界）を生きていることを指摘している。 トマセロ (2022/2023) はこの点を踏まえ，「生物が持つ知覚の観点からすると，生態的ニッチは，自己の経験的ニッチでもある」(p.61)と述べており，複雑な社会の中では状況認知を変化させるさまざまな経験がそれぞれの生物種が生き抜くための基盤となる。これは現代の学習主体においても，自己の経験を豊かにすることが動的なシステムを動かす原動力になると考えられる。さまざまな経験がさまざまな形で状況認知を変化させ，環世界を再構築していく，この動的なシステムこそが「経験・生態的学習環境」である。

### 4-2 学習環境エンジニア

前節では，生物の進化と同様に学習環境を動的なシステムである「経験・生態的学習環境」として考えていく必要性を述べた。本節では，その学習環境の構築において動的なシステムである経験・生態的学習環境をどのように創り出していくかについて検討する。

まず再度，生物界の話から入りたい。生物界の中には自身の生息域の環境を積極的に変化させ，その変化によって他の生物にも直接・間接的に影響を与える生物（ビーバーなど）がいる。このような生物は「生態系エンジニア」と呼ばれる。生態系エンジニアと呼ばれる生物は自身がよりよく生きるために自身の環境を変化させるということが根幹にあるが，自身が存在する生息域全体の環境に変化を与え，それによって他生物が利用する資源にも変化を生み，生息域全体を豊かにする。つまり，「生態系エンジニア」が自身の環世界においてよりよく生きようとすることが，他の生物にとっての未知の経験を創り出し，その未知の経験を通して生息域のさまざまな生物は状況認知を変化させ，その結果，生息域全体の豊かさを創り出していく。

これは学びにおいても同様である。「経験・生態的学習環境」では学習主体自身の経験を豊かにすることが動的なシステムを動かす原動力であることを述べた。学習主体が積極的に学びに関する状況認知を変化させ，それにより学習主体および学習主体の環境が相互作用しながら再構築されていく。この時の学習主体が「学習環境エンジニア」である。また，この学習主体を複数人として捉えると，さらに複雑なシステムとして動き出す。学習主体が自身の経験を豊かにする際に，そこに他者の存在を措定し，他者との関係の中で積極的に学びに関する状況認知に変化を起こしていく。すると，そこに関わる他者の環境にも変化が起き，結果的に学習主体，学習主体の環境，学習主体と共に生きる他者（=他の学習主体），他者の環境という4つのシステムが相互作用をしながら協働的に再構築されていく。学習環境エンジニアには誰もがなり得るが，日本語教育の文脈で第一に想起されるのは日本語学習者であろう。また，学びに関する状況認知に対して積極的に変化を起こすという点では，学習者の学習環境をより良いものへと変化させていこうとする意志を持つ「日本語教師」も「学習環境エンジニア」として機能する可能性を十分に持っていると言える。では，学習環境エンジニアの役割である経験を豊かにするとはどういうことであろうか。

### 4-3 学習環境エンリッチメント

4-1 節で述べたように，行動の意思決定は生物の目標に応じて決まり，その際の注意の対象となるのは自己の目標や行動に関連する状況である。つまり，人は何かしらの目的を持って計画・行動をし，その予見または行動を行った結果に対するフィードバックを得ながら，自己の行動を環境と共に調整して行っている。 トマセロ (2022/2023) はこれを「フィードバック制御システム」と呼んでいる。この「フィードバック制御システム」によって，「その都度の状況のもとで何が機能し何が機能していないかを決定して，知覚によるフィードバックを介して行動中に計画を変更したり，遠い未来のために学習したりすることが可能」 (pp.103-104) になる。つまり，主体が行動を起こすことにより環境に変化をもたらし，その結果，何かが起こるという環境との間の因果関係を捉えるということが重要な学びの一つである。これは ヴィゴツキー(1930-31/2005) が行動形態の主要な特質として述べた「刺激Aは，反応を呼び起こす。その反応というのは，B 点に作用を及ぼす刺激 X を発見することにある。このようにして，A 点と B 点との結合は，直接的にではなく，間接的に確立される。」 (pp.139-140) と一致する。つまり，学習主体 (A) はある目的 (B) のために，環境 (X) を道具的に利用して目的 (B) の達成を試みる。その際，単に環境 (X) を媒介するだけではなく，そこにはトマセロが述べるフィードバックを行うためのメタ的な視点があり，そのメタ的な視点で得た情報（フィードバック）を基に学習主体 (A) が環境 (X) との調整を行っていると考えられる。その意味では環境を目的の遂行のための道具として活用すると同時に，目的に向かっての学習を環境とともに行なっていると考えることができる（ 図2）。

**図2.**
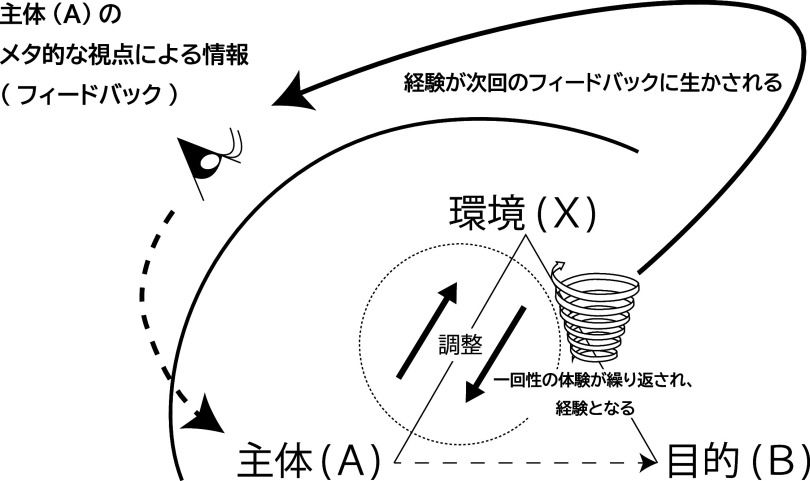
学習主体と環境の適応モデル.

この点について， 加藤・有元 (2001) も「リソースはそれを可視化する特定の文脈にあってはじめてリソースと捉えることが可能になるものであり，それが同時に特定のリソースを利用するような文脈を相互的・その場的に構成する」(p.11)と述べている。つまり，イメージ情報としてのメタ的な視点でニーズを明確にし，調整において具体的に実行できるのかという試行としての一回性の体験を繰り返すこと（経験）になる（ 槙，2004）。この繰り返しにより学習主体は学習環境に適応していく（ 図2）のであるが， ローレンツ(1973/2017) が「適応とは生物とその環境世界との間に存在する転移情報の増大であり，この増大は生物の内部における諸プロセスによって起こされるのであって，そのさい環境世界には目だった変化はない」(p.57)と述べたように，さまざまな体験・経験を積み重ねていく適応は環境自体を変化させるのではなく，環境にある異質性などの情報を「経験の中に持ち込む」必要があり，その経験を通して自分の中に多様性を持つことが新たな反省的思考につながる。では，学習主体 (A) が「学習環境エンジニア」として環境にある情報を経験の中に持ち込み，豊かにしていくにはどうすればよいであろうか。

動物園学では環境エンリッチメント (Environmental Enrichment) を重視した取り組みが行われている。環境エンリッチメントとは「動物を取り巻く環境を変化させることで動物の行動を豊かにしていこう」（ 細田，2014, p.169）とするもので，「飼育下に置かれている動物の環境に対し，追加あるいは変更を加えて野生での自然な行動を引き起こし，それが動物福祉につながるという考え」（ 細田，2014, p.169）である。そして， 伊藤ほか (2023) では ホージーほか (2009/2011) を参考に，この環境エンリッチメントの中で日本語教育分野においても応用可能性のあるものとして，以下の 4 つのエンリッチメントを挙げている。

a) 空間エンリッチメント:環境の構築的変化。遊具など自由に操作できるものの提供。
b) 感覚エンリッチメント:視覚，聴覚など感覚を刺激するものの提供。
c) 社会的エンリッチメント:他者とのかかわりのあるものの提供。
d) 認知エンリッチメント:知性を刺激するもの。問題解決に複雑さが伴うものの提供。
                             （ 伊藤ほか，2023, p.12）

そして， 伊藤ほか (2023) はこの4つの環境エンリッチメントの方向性である，自由に使えるリソース（空間的エンリッチメント），五感に訴えかけるリソース（感覚的エンリッチメント），他者との関わりを生み出せるリソース（社会的エンリッチメント），複雑さを伴う知的刺激のあるリソース（認知的エンリッチメント）は，我々が暮らす社会自体に潜在的に含まれているとも述べている。しかし，この指摘は潜在的に含まれてはいるが，それが顕在的になっていない可能性も同時に示唆されている。ここに環境エンリッチメントと「アフォーダンス」（ ギブソン，1979/1985）の関連性を捉えることができる。 ギブソン (1979/1985) はアフォーダンスについて「環境のアフォーダンスとは，環境が動物に 提供する (offers) もの，良いものであれ悪いものであれ， 用意したり供えたりする (provide or furnishe) ものである。」（p.137 下線は引用元による）と述べており，認知主体はこの環境から提供された情報を一部を知覚し，行動を選択するとされる。そのため，アフォーダンス自体は認知主体が知覚していても知覚していなくても，そこに存在するものである。さらに， ノーマン (2010/2011) はこのアフォーダンスのうち，認知主体に知覚されたアフォーダンスを「シグニファイア」と呼び，物理的，社会的世界で意味あるものとして解釈できるシグナルの重要性を述べている。つまり，環境から提供された情報（アフォーダンス）から学習主体にとって「意味のある価値づけ」（シグニファイア）を適切に環境エンリッチメントとして配置することで，学習環境エンジニアは学習主体の経験を豊かにすることができるのである。

## 5. 経験・生態的学習環境における意味のある価値づけ

4 節で述べてきたように，動的なシステムである経験・生態的学習環境の中で生きる学習主体に対する学習環境エンジニア（学習主体と同一人物/他者どちらも含む）の役割は，環境のアフォーダンスから学習主体にとってシグニファイアとなる「意味のある価値づけ」を適切に環境エンリッチメントとして配置することである。それにより，学びに関する状況認知に変化を起こし，学習主体および学習主体の環境に変化・再構築を促していくことである。つまり，重要な鍵となるのは学習主体が存在する環境における「意味のある価値づけ」である。この「意味」について， 岡崎 (2009) が重要な定義を提示している。

岡崎 (2009) は急速に変動する世界の中で自分自身が使い慣れていることばであっても，自身の生活をベースにすると，そのことばが意味することに対する想像力が及ばなくなることを「ことばの形骸化」，そして，それがより極大化した状態を「ことばの融解」と呼んでいる。そして， 岡崎 (2009) はことばの形骸化が進んだ例として，文字情報（例えば「貧困」など）を読む際に文字面だけで読んでいると，想像力を働かせて読まなくなり，情報の向こうにいる人，そこで起こっているコト，そこにあるモノを再生しながら世界を組み立てて読む（空腹から明日への希望が生まれることもなく，家族や自分自身が明日死ぬかもしれない不安や恐怖に打ちひしがれている状況を感じる）ということがなくなり，自分の生活経験の範囲内だけの理解（空腹で苦しんで大変だ，など）で文字情報を処理し，同時にその狭い範囲内で自己のアイデンティティーが形作られてしまうことを挙げている。この「ことばの形骸化/融解」は，技能実習生の実態が「低賃金」「長時間労働」「暴力」「失踪」「人権侵害」などの言葉とともに伝えられていても，その改善が国際的な批判を浴びるまでなかなか進められてこなかった状況からも理解できるであろう。そして，これは「ことばの側の問題，ことばの中の何かの要因の問題ではない。私たちと世界のつながりの問題に由来する問題である。」(p.36)と 岡崎 (2009) は指摘し，世界とのつながりは「自己を世界の中に位置付けて考えること」（ 岡崎，2009, p.78），「『自分にとって，世界はどうなっているか』という問いに応ずる形で形作られていくもの」（ 岡崎，2009, p.78）であるとしている。この点を広く捉えると言葉だけではなく，体験感覚が乏しく，経験への想像力が及ばない「体験の形骸化」とも言える。さらにもう一つの重要な側面は「他の人の視座も，自分の視座の中に保持して世界を見る」（ 岡崎，2009, p.78）という「『自分には見えていない世界』が実在していることを認識して『世界』を見る」（ 岡崎，2009, p.79）ことであると述べている。これは 4-1節で述べた生物種が自身を取り巻く環境から主観的に意味を構築して創り出して見ている世界「環世界」への想像力である。そして，このようにして想像力を働かせて「自己の生き方（=生態）と，対象（世界，自然界のコト・モノ・人）とのつながりを，自己を起点として紡ぐとき」（ 岡崎，2009, p.42）に「意味」は発生するとしている。つまり，環境から提供された情報（アフォーダンス）から学習主体にとって「自分自身と現在の環境はどのようなつながりがあり，何が起こっているのか」という意味を獲得する必要があり，そのために学習環境エンジニアは環境エンリッチメントとして「自分自身と現在の環境はどのようなつながりがあり，何が起こっているのか」，そして「今，何をすべきなのか」ということを考える基盤を配置することで，学習主体の経験を豊かにし，個別の学習環境を豊かにしていくことができるのである。さらにここで， 4-2 節で述べたように他者の存在を措定することでより豊かな経験・生態的学習環境の層を作り出すこともできる。その際に組み合わされる経験は単純に経験 A と経験 B の組み合わせから作られるものだけではなく，個別の経験 A や経験Bにはまったく存在しなかった段階としての異なる経験 C を得ることにつながる。つまり，経験同士が組み合わさることも一回性の出来事であり，その文脈において相互的・その場的に構成されるものであり，その経験が何層にも重なり合い，経験・生態的学習環境は持続的により大きく，より豊かなものとして創られていく（ 図3）。

**図3.**
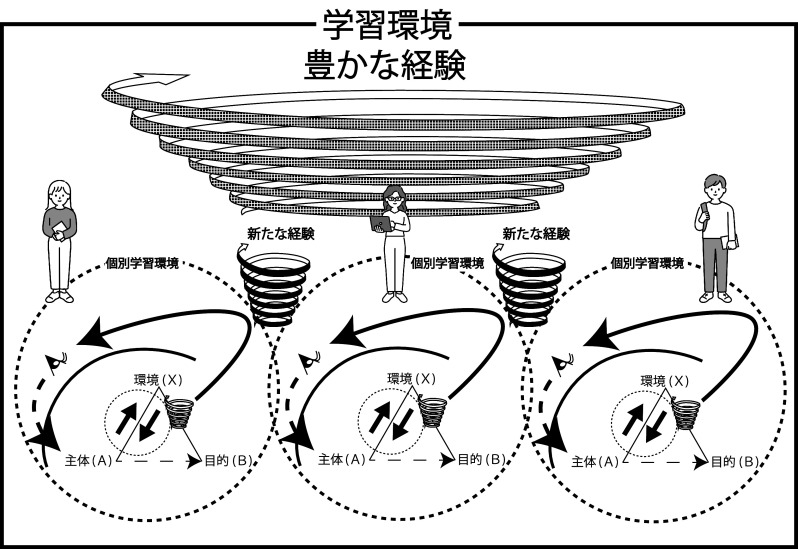
経験・生態的学習環境.

## 6. まとめと展望

本稿では，先行研究の「学習環境」という用語の使用実態を調査し，従来の学習環境は研究者が静的な学習者を中心にして，その特徴を捉えた「形態特徴的学習環境」が「学習環境」とされていることを述べた。しかしながら，人間や社会は絶えず変化し，学びにおいても学習主体は環境と相互作用をしながら変化していく能動的な循環関係であることから，動的な学習者/学習環境の姿を捉えようとする「経験・生態的学習環境」へと「学習環境」の捉え直しをしていくことを主張した。

第 1 節でも述べたように，従来の学習環境は日本国内/日本国外，漢字圏/非漢字圏，教室環境/自然環境，対面環境/オンライン環境のように「形態特徴的学習環境」として捉えられてきた。また，このように触れられる学習環境は一方の優位性や不十分さを述べるレトリックとして使用され，そこには発信者による暗黙の学習環境に対する上下関係が作り出されやすい。本稿では，この形態特徴的学習環境の捉えに対して，動的システムとしての「経験・生態的学習環境」を提示し，学習環境に上下関係はなく，その場でその場で学習主体と環境との調整によって作り出されるものであることを述べた。また，このような経験・生態的学習環境を構築していくためには，学習環境エンジニアが「自分自身と現在の環境はどのようなつながりがあり，何が起こっているのか」，そして「今，何をすべきなのか」という意味のある価値づけとなる環境エンリッチメントを配置していくことで学習主体の経験および学習環境を豊かにしていくことができること，さらにはそれをコミュニティとして構築していくことで持続的かつより豊かな環境となっていくことを述べた。

一方で，本研究にも限界がないわけではない。特に本研究は従来の学習環境の捉えに関しては先行研究に対する調査で示せたものの，新たな捉えである経験・生態的学習環境について具体的なデータで示すことはできていない。ただしこれは，経験・生態的学習環境は個別の文脈に沿い，その場，その場の文脈で構築されるものであることから本研究が不十分であることを示すものではない。むしろ今後，本研究で示したモデルから経験・生態的学習環境の実態に関するデータを蓄積していくことで具体的かつリアルな学習者の経験・生態的学習環境が明らかになっていくものであると考える。近年，医学において従来の「How（どのような仕組みで病気になるのか）」から「Why（なぜ病気になるのか）」という根源的な問いに迫る「進化医学」が注目を集めている（ 長谷川，2020）。日本語教育においても「なぜ日本語を教える/学ぶのか」という「進化日本語教育学」を想定した時に，それぞれの学習主体が持つ個別学習環境に迫る研究は言語的なスキルを身につけることだけではなく，日本語を学ぶことが人生においてどのような意味を持つのか，を明らかにしていく重要な研究となる。そのような研究に対して本研究のモデルが一助となるように今後も研究を深めていきたい。

## Data Availability

Dataverse: List of surveyed references on “learning environment” in Japanese language education. https://doi.org/10.7910/DVN/FENP9V ( Ito, 2024). 本プロジェクトは以下の基礎データを含む:
• List of surveyed references on learning environment in Japanese language education.pdf データは クリエイティブ・コモンズ・ゼロの “No rights reserved” 著作権放棄条項 (CC0 1.0 Public domain dedication) に則って入手することができます。

## Footnotes

^1^
実際の抽出は 2023 年 9 月より順次，論文の閲読調査を行い，最終的に 2024 年 4 月 2 日に抽出を完了した時点で，検索条件に沿って抽出したものとそれまでに抽出した結果に相違がないことを確認した。

^2^
ユクスキュルの提示した「Umwelt」を「環境世界」または単に「環境」と訳している文献もあるが，本文献の訳者である日高は「環世界」と訳している。これについて日高は客観的な「環境とUmweltとは対立するものとユクスキュルはとらえている」(p.165)「環境という語を含む「環境世界」はUmweltの訳語としては適切ではないと思い，「環世界」という語に変えることにした」(p.165)と述べており，本稿もこれに従う。
