# Supplementary material for: Re-conceptualization of the learning environment as a dynamic system
Source: F1000Res. 2024 Oct 10;13:1187. [Article in Japanese] [Version 1] doi: 10.12688/f1000research.153938.1 (PMC11586609; doi:10.12688/f1000research.153938.1)
Supplement: Peer review history

# Reviewer report: Reviewer response for version 1

**DOI:** 10.5256/f1000research.168900.r330851

**Published:** 2024-12-17

**Authors:** Satomi Saito

**Affiliations:** 1 Tokyo University of Foreign Studies, Tokyo, Japan

論文：学習環境を動的なシステムとしてとらえ直す

 【１】全体へのコメント

 　どのような学習環境が教育により良い効果をもたらすかは重要なテーマでありながら、これまで観点があいまいでした。そこに着眼し、これまでの論文から学習環境に対する認識を詳細に分析している点が大変良いと思いました。

 　従来の静的な学習環境を「形態特徴的学習環境」と位置づけ、今後の学習環境のあり方を動的なシステムとして捉え直す必要があるとする主張は、学習者による主体的な学びを目指す現在の教育を斬新な視点から強く支持するものだと考えます。

 【２】良い点

 １．調査概要が十分に再現可能な方法であり、良いです。

 ２.  「学習者の周りに配置するリソースとしての学習環境」について、このようなリソースがいくら整っていてもそれにアクセスできる空間・時間・手段・学習者の状態などが十分でなければ学習環境が整っているとは言えないと考えられます。学習環境を動的に捉え直す視点はこれらリソースと学習者を結び直すことができるのではないでしょうか。こうした視点を展示する筆者の主張に今後の日本語教育のみならず教育のあり方に対する新しい提案の可能性を感じました。

 ３．「生態系エンジニア」が環境に影響を与え、周囲全体を豊かにするという例を興味深く拝読しました。「学習環境エンジニア」についてのイメージを持つことができました。

 ４．環境エンリッチメントの4つの観点の提示は、学習環境を考える際の具体的な手掛かりとなるものとして興味深いです。さらにこれを、静的な学習環境を動的なものに変えるために意識的に取り入れるのがよいと明確に主張してはどうでしょうか。

 【３】説明を求めたい点

 １．筆者が「学習環境」の重要性をどのように捉え、分析にいたったのか伺いたいです。

 ２．日本語教育における「学習環境」をとらえ直すための4観点が、どのようにして設定されたのか明らかにしていただきたいです。筆者のこれまでの著述を含めた先行研究からのものなのか、本論の文献調査のなかで筆者が設定したものなのか明示していただけるとよいです。

 ３．「学習環境エンジニア」の記述の部分で「日本語教師も学習環境エンジニアとして機能する可能性を持っている」という部分がありますが、日本語教師は学習者にとってリソースの一つであるという考え方もあると思います。その点を整理して論じていただけると、主張に説得力が高まると考えます。

 ４．まとめで筆者も述べられていることですが、「経験・生態学的学習環境」についてどのようなものか、実際の日本語教育においてどのように実践されていくものなのか具体的な説明があったらと思いました。たとえば、「環境から提供された情報から、学習主体にとって意味のある価値づけ(シグニファイア)を適切に環境エンリッチメントとして配置することで、学習環境エンジニアは学習主体としての経験を豊かにすることができる」との主張について、学習者が「実際の学習環境で」、「どのようなふるまいをして」「これらを実現する」のか、ぜひ伺いたいです。

（該当する場合は要回答）統計分析および解釈は適切ですか。

対象外（統計を使っていない）

本研究は明確かつ正確に提示されたものであり、最新の文献を引用していますか。

はい

方法と分析について第三者による再現が可能となるよう十分な詳細が提示されていますか。

はい

結果の基礎となるソースデータはすべて入手可能で再現性を十全に保証していますか。

はい

結論は結果により妥当な裏付けを得ていますか。

はい

研究設計は適切で学術的価値がありますか。

はい

Reviewer Expertise:

日本語教育　外国にルーツのある高校に対する日本語教育　コーパス　語彙学習

I confirm that I have read this submission and believe that I have an appropriate level of expertise to confirm that it is of an acceptable scientific standard.

# Reviewer report: Reviewer response for version 1

**DOI:** 10.5256/f1000research.168900.r332479

**Published:** 2024-11-25

**Authors:** Hideyuki Suzuki

**Affiliations:** 1 Ibaraki University, Mito, Japan

＜評価できる点＞

 学習環境という定義が曖昧な概念に注目し，日本語教育研究の文脈において，この言葉がどのような意味で使われているのかを調査した結果として，従来の学習環境の捉え方が「形態特徴的学習環境」であることを指摘した点．特に，４つの形態が歴史的な段階ではなく，全時代において存在する捉え方であることを証拠をもって明らかにした点は興味深いと思います．

 上記の把握に基づいて提案される「経験・生態的学習環境」という考え方は，一見，既に存在しているヴィゴツキーの道具による媒介や活動理論の焼き直しに見えるものの，そこに環境エンリッチメントという概念を導入することで，学習の可能性を（多分，活動理論よりも積極的に）デザインする道筋を示しており，この点で教育研究に与える貢献が大きいと判断します．

 「豊かな経験」という教育（学校）言説に取り込まれがちな用語を曖昧なままに使わずに，理論的にクリアにしようと試みている点を高く評価します．

 ＜加筆修正のお願い＞

 【日本語教育という研究設定は何だったのか】

 本論文は，日本語教育の領域で「学習環境」という言葉の使用を分析し，そこから学習環境の動的な捉え方を提示するという流れになっています．しかし，動的学習環境の提案の段階で，日本語教育との関連が弱くなるため戸惑います．学習理論一般に対して提言をするのであれば，前半のレビューを日本語教育に限定する必要性はないと思われますし，少なくとも限定する学術上の根拠を示す必要があります．もしくは，動的学習環境が日本語教育にこれまでなかったどのような発展をもたらすのか述べるべきかと考えます．

 【生態的学習環境の学術的位置づけ】

 本論文では，日本語教育における学習環境は形態特徴的に捉えられてきたという現状把握を前提として，経験・生態的学習環境という考え方が示されています．言語教育（日本語教育も含む）の分野では，生態学的言語論に基づく日本語教育研究があります（宇都宮2013, 岡崎2008など）．これらは，言語を創発的に捉え，それ故に言語獲得を関係性の中に捉えるというものだと理解します．本論文の経験・生態的学習環境や学習環境エンジニアといった考え方と全く同じではないものの，考え方の根幹には共有するものがあるかと思います．「日本語教育　and 学習環境」というサーチからは洩れるかもしれませんが，日本語教育の領域において，このような先行例がある以上，これらを批判的に評価した上で，それとの比較において今回の提案を示すべきではないかと考えます．

 【「自ら作り出すものとしての学習環境」に含まれる活動理論の取り扱いについて】

 形態特徴的学習環境の特徴として，学習者と周りの相互作用の見落とし，学習者の非アクター的捉え，を挙げていますが，「自ら作り出すものとしての学習環境」の例としてあげられている活動理論は，形態特徴的学習環境には当たらないように思われます．経験・生態的学習環境の提案の中で示される環境適応モデル（図２）は，明らかにヴィゴツキーの３角形に基づいており，そう考えれば，方向は違うかもしれませんが，ヴィゴツキーの３角形の拡張ともいえる活動理論を，形態特徴的学習環境の例にいれるのは，活動理論を過小評価するものではないでしょうか．従来の学習環境の捉え方を否定した上で，新しい捉え方である「経験・生態的学習環境」を提案するという流れの中で，活動理論が前者に入れられて処理されてしまっていることに違和感があります．経験・生態的学習環境と活動理論は全く連続性がないのか，活動理論の何らかの問題を乗り越えるものとして提案されているのか，等の説明が必要かと思います．この点についての修正，もしくは追加説明をお願いいたします．

 ＜できればご検討いただきたいこと＞

 【レビューにおける学習環境の分類に関して】

 ４つのカテゴリーに分けていますが，各カテゴリーの基準の明示が必要かと思います．例えば「学習の物理的な場」の中には，日本語との接触機会等，学習環境を「リソース配置」と捉えているような事例が含まれているように見えます．このような読者側の混乱を防ぐために，各カテゴリーに何が入って，何が入らないのかという基準を示されるとよいと思います．

（該当する場合は要回答）統計分析および解釈は適切ですか。

対象外（統計を使っていない）

本研究は明確かつ正確に提示されたものであり、最新の文献を引用していますか。

はい

方法と分析について第三者による再現が可能となるよう十分な詳細が提示されていますか。

はい

結果の基礎となるソースデータはすべて入手可能で再現性を十全に保証していますか。

はい

結論は結果により妥当な裏付けを得ていますか。

はい

研究設計は適切で学術的価値がありますか。

一部該当

Reviewer Expertise:

教育工学，認知科学

I confirm that I have read this submission and believe that I have an appropriate level of expertise to confirm that it is of an acceptable scientific standard, however I have significant reservations, as outlined above.

# Reviewer report: Reviewer response for version 1

**DOI:** 10.5256/f1000research.168900.r330843

**Published:** 2024-11-05

**Authors:** Jihyun Yoon

**Affiliations:** 1 Chuo University, Tokyo, Japan

**【評価できる点】**

 　本稿は、日本語教育における「学習環境」の再定義を試みたものです。従来の「学習環境」の概念は静的なもので、学習者を中心とした形態的特徴であると捉える傾向がありました。しかし本稿の著者は、学習者と環境が相互作用しながら変化する「経験・生態的学習環境」への転換を提唱しています。この新たな視点では、学習環境が学習者の経験を豊かにする重要な要素として位置づけられ、学習環境をデザイン・調整する「学習環境エンジニア」の役割が不可欠です。また、このような視点を持つことによって、様々な人々の経験を融合させ、より持続可能で豊かな学習環境を構築できるとしています​。　

 　本稿における研究成果の高く評価できる点として、主に以下のような特徴が挙げられます。

 　第一に、学習環境の再定義における先進的な視点です。本稿では、学習環境を「静的な場」から「動的なシステム」として捉え直し、学習者と環境が相互作用を通じて変化する「経験・生態的学習環境」を提唱しています。学習環境を動的なプロセスと見なし、学習者の主体的な成長を重視することで、学習環境のデザインに関する新たな枠組み、ならびに教育研究に新しい方向性を示しています。

 　第二に、学習環境エンジニアという新しい概念の提案です。学習環境エンジニアの役割を提案し、学習者が学びに集中できる環境を積極的に整える必要性を説いている点も独創性のある視点といえます。これにより、教育者や管理者が学習環境の面で果たすべき役割が明確化され、学習環境の改善における実践的な指針が提示されています。

 　第三に、エンリッチメントの多様な要素の導入です。環境エンリッチメントの要素として、感覚、空間、認知、社会的エンリッチメントを取り入れることで、学習環境を多角的に充実させる手法が提案されています。特に、動物行動学の環境エンリッチメントの概念を学習環境に応用するアイデアは斬新で、学習者の多様なニーズに対応する実用的なアプローチとして評価できます

 　第四に、学習環境における持続可能性と協働の重要性の強調です。学習環境を「持続的かつ豊かなもの」として構築するため、個々の学習経験を組み合わせることの重要性が強調されています。これにより、学習環境が個別の学習者を超えてコミュニティ全体の成長に寄与することが明確化され、教育現場における協働の意義を再認識させています。

 　以上のように、本稿で提唱されている「経験・生態的学習環境」の概念は、従来の日本語教育の枠組みに囚われず、新しい観点から学習環境を見直す試みであるため、日本語教育分野のみならず、教育理論全般にも革新的な貢献が期待されます。

**【加筆が必要な点】**

 まず、具体的なデータの補強が求められます。「経験・生態的学習環境」モデルの提案は理論的に重要ですが、具体的な事例等の提示が十分とは言えません。

 　次に、「学習環境エンリッチメント」について、どのように実装するかという実践例の記述がありません。例えば、感覚エンリッチメントや社会的エンリッチメントの具体的な設計、導入プロセス、評価方法等を示すことによって、読者はその概念をより正確に理解できると思います。

 　最後に、「学習環境エンジニアの役割」の具体化が望まれます。その重要性は述べられていますが、具体的にどのようなスキルや知識が必要なのかが曖昧です。求められる役割やスキル等についても詳述することで、各教育現場での導入・応用の可能性が一層高まると考えられます。

（該当する場合は要回答）統計分析および解釈は適切ですか。

対象外（統計を使っていない）

本研究は明確かつ正確に提示されたものであり、最新の文献を引用していますか。

はい

方法と分析について第三者による再現が可能となるよう十分な詳細が提示されていますか。

はい

結果の基礎となるソースデータはすべて入手可能で再現性を十全に保証していますか。

はい

結論は結果により妥当な裏付けを得ていますか。

はい

研究設計は適切で学術的価値がありますか。

はい

Reviewer Expertise:

日本語教育学・応用言語学

I confirm that I have read this submission and believe that I have an appropriate level of expertise to confirm that it is of an acceptable scientific standard.

# Reviewer report: Reviewer response for version 1

**DOI:** 10.5256/f1000research.168900.r330848

**Published:** 2024-10-25

**Authors:** Tomoko Okimoto

**Affiliations:** 1 Tokyo University of Foreign Studies, Tokyo, Japan

論文：学習環境を動的なシステムとして捉え直す

 【１】全体へのコメント

 　従来の「形態特徴的学習環境」から、学習主体と環境が相互作用することで学習環境が変化する動的な「経験・生態的学習環境」への転換を提唱している点が斬新であり、日本語教育の学習場面で学習主体の経験と環境との関係性を動的に捉え直すという試みは、学びの本質に迫っており、教育理論としても革新的です。

 　また、本稿における学習環境をコミュニティとして構築していく点、また図3の学習環境の解説から、学習者が学習を続ける過程で生じる時間的・空間的移動に影響されることなく、学習主体によりコミュニティが新たに構築され続け未来へと繋がるイメージが提案されていると考えられます。

 【２】加筆するとよいと考えられる点

 １．具体的な実践例について

 　本稿でも述べられているように、経験・生態的学習環境について具体的なデータがありませんが、著者も記述しているように、今後の研究調査によるデータ蓄積が待たれます。

 ２．学習者の多様性への配慮

 　本稿では学習主体と環境の相互作用について論じていますが、学習主体の多様なニーズ・個々の認知能力・個別の背景などに関する具体的な考察がありません。具体例を１つ記述するとより本稿への理解が深まるかと考えられます。

 ３．評価方法や効果測定について

 　経験・生態的学習環境において、学習主体の経験の豊かさ、個別の学習環境の豊かさの評価、シグニファイアの適切な配置に対する効果測定について議論し具体例を示すと、理論が実践に結び付きやすくなるかと考えられられます。

 4.他分野との統合的視点

 　既に生物学からの応用、医学への連携（進化医学）など、他分野との統合的視点が述べられているので、更に後述のように（【３】より詳しい説明を求める点）、学習主体の認知についての追記など、認知科学・社会学などの理論や方法論を取り入れるとより説得力のある説明となると考えられます。

 ５．教育者への具体的な支援策

 　後述のように（【３】より詳しい説明を求める点）「学習主体」と「学習環境エンジニア」については、使用場面に応じた書き分けが必要と思われますが、「学習環境エンジニア」の中でも「日本語教師」がシグニファイアを適切に「選択し」、環境エンリッチメントとして配置する方法について必要な支援（トレーニング・リソース提供など）について明示的に提案すると、本稿による提唱理論が教育現場で実行可能かと考えられます。

 【３】より詳しい説明を求める点

 １．動機について

 　学習環境を再検討するに至った、本稿執筆の動機が加筆されるとよいかと思います。

 ２．4つの観点について

 　「２．本研究で行った調査概要」（Page3）にある4つの観点（「学習を行う物理的な場」「学習者の周りに配置するリソース」「授業内で行う学習活動の多様性，心理的側面への配慮」「自らが作り出すもの」）は、何を基準に作成されましたか。例えば先行研究の論文を分析し、項目ごとに分類している際に、大項目として名付けた、などでしょうか。

 ３．観点「自らが作り出すものとしての学習環境」について

 　「3-4　自らが作り出すものとしての学習環境」（page5）は、それまでの3つの観点の包括的内容であり、学習者による自主的前進と考えられますが、「動的・生態的学習環境」の１つとして捉えられるのではないでしょうか。

 ４．想定場面について

 　「4-2　学習環境エンジニア」の節で述べられている、「自分の発展と共に周りの環境を変化させる機能」について、このような相互作用は学びの場に適用したとき、対面（学習主体と教師、学習主体同士が直に会う）場面なのか、オンライン（直接会わず、間に何らかのツールまたはリソースを介す）場面なのかは、想定されていますか。

 ５．フィードバックについて

 　「4-3学習環境エンリッチメント」で引用されている「フィードバック制御システム」は自己認知によるフィードバックなのか、他者からの言語・反応によるフィードバックなのか、説明があった方がよいかと思います。

 ６．学習主体のメタ的視点について

 　「4-3学習環境エンリッチメント」での主張として「学習主体がある目的のために、環境を道具的に利用して目的の達成を試みる。その際、メタ視点で得た情報をもとに学習主体が環境との調整を行っている」は大変分かりやすく俯瞰した、学習主体の行動観察と考えられます。しかしながら、この「メタ的視点で情報を得る」ことは、個々の学習主体の認知度にも関わってくるのではないでしょうか。グラデーションのある学習主体の認知度についても言及および考察があった方がよいかと思います。

 ７．「生態系エンジニア」と「学習環境エンジニア」の違いについて

 　「4-3学習環境エンリッチメント」（page9, L13から）の「環境から提供された情報から・・・」で述べられているように、「学習環境エンジニア」がシグニファイアを適切に「選択し」、環境エンリッチメントとして配置するということは、「学習環境エンジニア」が恣意的に配置していると考えられます。「学習環境エンジニア」の原型である「生態系エンジニア」（生物）が恣意的に環境へ影響しているとは恐らく証明されていないと考えられますので、「学習環境エンジニア」と「生態系エンジニア」の違いの説明を入れるとより論文への理解が深まるかと思います。

 ８．「学習主体」と「学習環境エンジニア」の書き分けについて

 　「５．経験・生態的学習環境における意味のある位置づけ」（page9, 下から9行目）の「環境から提供された情報から・・・」では「学習環境エンジニア」が想像力を働かせ、「意味」に気づき、適切に基盤を配置する必要性がまとめてありますが、本来同一である「学習主体」と「学習環境エンジニア」（page7）が文中に交互に役割分担して出現するため、「学習者」と「教師」の関係性として読み取れます。ここで「学習環境エンジニア」を「教師（リードする側）」として説明に使用しているのであれば、page7にあるように「日本語教師＝学習環境エンジニアとして機能する」点を、再度確認するとよいのではないでしょうか。

 ９． 「学習環境エンジニア」の「気づき」について

 　8と同様の箇所ですが、「５．経験・生態的学習環境における意味のある位置づけ」（page9, 下から9行目）の「環境から提供された情報から・・・」では「学習環境エンジニア」が想像力を働かせ、「意味」に気づき、適切に基盤を配置する必要性がまとめてありますが、「学習環境エンジニア」の気づきと配置が行われることが前提になっています。「学習環境エンジニア」の気づきのレベルまたは確実性について記述を加えるとよいのではないでしょうか。

 １０． 従来の学習環境への言及について

 　「６．まとめと展望」の第二段落にある先行研究から分析した学習環境について「発信者による暗黙の学習環境に対する上下関係が作り出されやすい」とありますが、４つの環境（物理的・リソース・心理的・創造的環境）がそれぞれ独立しており、双方向の影響もないことから、「上下関係」ではなく「カテゴライズした学習環境の存在有無」に当たるのではないでしょうか。

 【４】些末な質問

 　「２．本研究で行った調査概要」内に、「（論文内で）具体的な記述があったものを中心に」（下から3行目）とありますが、具体的な記述があった論文は何本かを追記すると読みやすいかと思いました。

 （拝読しながら疑問に思った質問です。ご放念頂いても大丈夫です。）

（該当する場合は要回答）統計分析および解釈は適切ですか。

対象外（統計を使っていない）

本研究は明確かつ正確に提示されたものであり、最新の文献を引用していますか。

はい

方法と分析について第三者による再現が可能となるよう十分な詳細が提示されていますか。

はい

結果の基礎となるソースデータはすべて入手可能で再現性を十全に保証していますか。

はい

結論は結果により妥当な裏付けを得ていますか。

はい

研究設計は適切で学術的価値がありますか。

はい

Reviewer Expertise:

日本語教育

I confirm that I have read this submission and believe that I have an appropriate level of expertise to confirm that it is of an acceptable scientific standard, however I have significant reservations, as outlined above.
